# Case of Congestive Fever, with Remarks

**Published:** 1839-09-21

**Authors:** John R. Buck


### *Case of Congestive Fever, with Remarks.* By JOHN R. BUCK, M. D.

To the Editors of the Medical Examiner.

GENTLEMEN,—In the month of October last I met with a well marked case of congestive fever, the history of which I have preserved,—and thinking it may not be uninteresting to some of your readers, I send you a detail of the symptoms and treatment. If you think it worthy an insertion in your valuable journal, it is at your service.

Very respectfully yours, &c.

J. R. BUCK.

*Memphis, Tenn.*, August 23, 1839.

I was requested, in October last, to see, in consultation with the medical attendant, Mr. H——, a middle aged man, in easy and comfortable circumstances, who had always lived temperately. He had been indisposed for several days, complaining of debility, anorexia, with a general feeling of malaise. The evening previous to my seeing him, he was taken with a very severe chill; his physician, who was immediately called in, prescribed sulphate quinine, calomel āā gr. xx.; which was repeated in half an hour, and again in an hour. The chill having somewhat abated, the calomel was discontinued, and the quinine reduced to ten grains, which was given every hour, for several hours, when the patient complained of great ringing in the ears. This, the physician hailed as the harbinger of a speedy cure,—and declared to me, upon my arrival there about two hours afterwards, that he had never, in any case, seen this symptom supervene, that a favourable change did not speedily follow. The patient, when I saw him, was lying in bed, with his head and shoulders elevated, an expression of alarm in his countenance, delirium rather active, skin cool and moist, pulse fifty-five, small and oppressed, respiration twenty-six in the minute. I proposed bloodletting, but neither physician nor friends agreeing with me, it was not adopted. The quinine was again given in small doses for four hours, when another chill came on, in which he died.

This I look upon as a well marked case of congestive fever, as it occurs in this climate. I do not consider it a disease, *sui generis*, but an aggravated or malignant intermittent. The history of the disease, as well as pathological anatomy, prove them to be identical: the same condition of pulse, skin, and respiratory organs, are observable in both diseases; dissection reveals nothing in the one, that is not observed (though not to the same extent) in the other. This fact I look upon as being very important in a practical point of view. It has been proven satisfactorily by Dr. McIntosh, that intermittent fever is not a disease of debility, and that bloodletting during the paroxysm, is not only safe, but in a large majority of cases, much the best method of relieving this troublesome disease. For what purpose, I would ask, are the enormous doses of quinine given in this disease, (congestive fever?) May not the extreme prostration which sometimes accompanies it, be owing in some measure to the herculean doses of this medicine? "All over-excitement is followed by a corresponding debility." If, then, we give a patient, whose system is already prostrated by disease, such over-doses of any stimulant, is it not reasonable to suppose that we will have a corresponding depression? It is affirmed by some, however, that quinine, in large doses, is not stimulant, but narcotic; as it is not my intention to discuss the medicinal properties of this medicine, I shall pass this remark over, or, for argument's sake, admit it. Is there any symptom in congestive fever indicating a narcotic? We have delirium, coolness of skin, small and oppressed pulse, hurried respiration, &c.; but does such a condition of things indicate a narcotic? On the contrary, does not the determination of blood to the head contra-indicate it? The professed object which most physicians in this country have in view, in giving these large doses of quinine, is not to produce a narcotic effect, but by diffusing the blood contained in the internal organs, relieve the congestion. If, then, the object in the treatment of this disease be to relieve congestion, our inquiry should be how this may be soonest and best done: that bloodletting is the most direct, as well as the safest method of relieving congestion in this disease, I am fully persuaded. These remarks have not been made as the result of long experience, or of a very extensive practice, but merely with a view of calling the attention of the profession to this interesting disease, which is considered by the physicians in this part of the country as almost an *opprobium medicorum.*

